# Precise genome modification of agrobacterium based on one-step homologous recombination via endogenous transfer DNA

**DOI:** 10.3389/abp.2026.16599

**Published:** 2026-05-25

**Authors:** Yu Liu, Geng Sun, Chunyan Gao, Rui Gao, Jian Tang, Menghu Wang, Jian Guo, Rui Guo

**Affiliations:** 1 School of Chinese Medicine, Bozhou University, Bozhou, China; 2 Key Laboratory of Chinese Medicine Materials Research of Anhui Higher Education Institutes, Bozhou, China

**Keywords:** agrobacterium, precise genome modification, sacb, T-DNA, VirD2

## Abstract

Here, a simple and precise genome modification method for agrobacterium was described and tested in strain GV3101. Taking the induced endogenous Transfer DNA (T-DNA) as the single-strand homologous repair template, this makes it possible to achieve valid and accurate genome modification for agrobacterium even without the strengthening of gene editing tools such as the CRISPR/Cas9 system. In addition, the introduction of tool plasmids into recipient agrobacteria can be achieved by the freeze-thaw method rather than complex triparental mating or expensive electroporation, which makes it more convenient. Here, the modification rate of agrobacterium strain GV3101 at both the upstream and downstream of VirD2 is 4.5% with 100% sequence accuracy. This research provides a valuable potential tool for genetic engineering in agrobacterium, with potential applications in biotechnology and agricultural sciences.

## Introduction

Agrobacterium is an engineered bacterium widely used in plant genetic transformation, with dozens of commonly used strains (Gelvin, 2017). Different strains with distinct genotypes and phenotypes are applied in plant genetic engineering research. However, the mechanisms of agrobacterium infection and T-DNA integration into the plant genome have not been fully deciphered (Gelvin, 2017; Tzfira et al., 2004). Different plants have different susceptibility to agrobacterium strains. Screening mutant strains and artificially creating specific genetically engineered strains are important ways to reveal the working mechanism of agrobacterium (Goralogia et al., 2025; Vergunst et al., 2005).

Using plasmids carrying suicide genes such as pK18mobSacB to perform gene editing of bacteria is a widely applied method (Chan et al., 2015; Peng et al., 2025; Zhou et al., 2025). In the routine modification process of agrobacterium, two rounds of homologous recombination are usually required. In the first round, antibiotic such as kanamycin is commonly used to force the tool plasmids, which lack the ability of autonomous replication in agrobacterium, integrate with the endogenous Ti or Ri plasmid in agrobacterium through homologous recombination, since the tool plasmids containing resistance genes cannot exist independently. In the second round, the SacB suicide gene in the tool plasmids is utilized for negative screening to select the products of the second homologous recombination that do not contain the tool plasmids. During these processes, a large number of unmodified agrobacterium strains are usually obtained, which results in very low overall modification efficiency (Xiang et al., 2001).

Here, we establish and describe a method that utilizes the self-produced T-DNA to assist in the precise modification of agrobacterium itself in detail for the first time. Different from traditional methods, it requires only one round of homologous recombination to achieve precise modification of agrobacterium. The introduction of tool plasmids into recipient agrobacteria can be achieved simply by the freeze-thaw method, without the need for triparental mating or electroporation, which could simplify the modification work.

VirD2 is a crucial protein in the agrobacterium-mediated genetic transformation process. It plays a pivotal role in the horizontal transfer of T-DNA from the bacterium to the plant cell and is essential for the successful integration of T-DNA into the host genome, making it a key component in genetic engineering and biotechnology applications involving agrobacterium (Gelvin, 2010; 2017). The modification of the VirD2 is crucial for the study of its gene function and is a key to creating integration-less agrobacterium strains (Lee et al., 2019). In our previous work, we had successfully modified the downstream of VirD2 gene in the agrobacterium strain GV3101 by deleting the 238 amino acids at the C-terminus and adding different transport peptide sequences derived from the VirF protein of the agrobacterium strain LBA4404, which can effectively transfer across cells via the type IV secretion system (T4SS). In this study, we optimized the modification process for agrobacterium, and attempted to precisely modify the upstream and downstream of specific genes synchronously.

## Materials and methods

### Equipments, materials and reagents

The core equipment, materials, and reagents used in this study are listed as follows.

PCR Thermal Cycler (Eastwin, ETC821DG); Gel Imaging System (Vilber, Quantum CX5);
Oscillating Incubator (ZhiCheng, ZWYC-2933); Super Clean Bench (OptiClean, 1300).
Competent cells of agrobacterium strain GV3101 (Self-made, applicable to the freeze-thaw method);
The plasmid pK18mobSacB was purchased from Bioflin;
The plasmid pBBR1MCS-2 was provided generously by Chunli Liu from Jiangnan University;
PCR-Kit, 2x Taq Mix C (FanTing, B103);
Ezmax® Universal CloneMix (TOLOBIO, #24305);
D5000 plus DNA Marker (Adamas life, E9071) with supplemental loading buffer (1X) and Gel-Red (8.3X);
LB medium (10 g/L tryptone, 5 g/L yeast extract, 10 g/L NaCl, pH = 5.5 or 7.0 as required);
6X DNA Loading buffer with Gel-Red (60 mM EDTA-Na_2_, 10 mM Tris, 40% (v/v) glycerol, 0.03% (w/v) Bromphenol Blue, pH = 8.0, supplied with 50X GelRed (Tsingke, TSJ003)).

### Plasmid construction

#### Backbone tool plasmid vector pSG-SacB05

**FIGURE 1:**
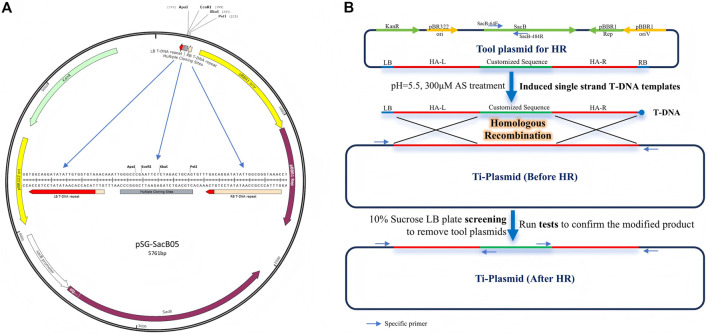
Brief map of backbone plasmid vector pSG-SacB05 **(A)** and schematic diagram for agrobacterium modification **(B)**. LB: left T-DNA repeat; RB: right T-DNA repeat; HA-L: left homology arm; HA-R: right homology arm.

The pBBR1 oriV and pBBR1 Rep sequences originated from pBBR1MCS-2, the SacB gene flanked by SacB promoter originated from pK18mobSacB were assembled with pBR322 ori, aminoglycoside phosphotransferase gene (Kan^R^), and the T-DNA repeats to form a complete plasmid using Ezmax® Universal CloneMix. The multiple cloning sites embedded in the left T-DNA repeat and the right T-DNA repeat could facilitate the insertion of template sequences. An additional pBR322 ori is beneficial for carrier construction, as pBBR1MCS-2 is a medium copy number plasmid, which leads to low yield during plasmid extraction. The SacB gene is a suicide gene when sucrose is present. A brief vector map of pSG-SacB05 can be found in Figure 1A.

#### Tool plasmid vector pSG-SacB10

The sequences of VirD1, VirD2, and VirD3 were amplified from agrobacterium strain GV3101, and the sequences of SpyTag, VirF, and VirF variant were directly chemically synthesized at Tsingke. The purified PCR product fragments of VirD2, SpyTag, VirF and VirD3 were assembled into *Xba* I and *Pst* I sites of pK18mobsacb using Ezmax® Universal CloneMix to make pSG-SacB01. Subsequently, this sequence combination was assembled into pSG-SacB05 backbone at *EcoR* I site to make pSG-SacB06-2G. In addition, pSG-SacB09 was constructed with VirF sequence replaced by VirF variant through the same processes. Then, the PCR products of VirD1 from GV3101, SpyTag, VirD2, and VirF-VirD3 from pSG-SacB06-2G were digested with restriction endonuclease *Apa* I, *Xba* I or *Bsa* I, in order to assemble them into *Apa* I and *Xba* I sites of pSG-SacB05 through Golden Gate Assembly to finally make pSG-SacB10.

### Modification Steps

The schematic for precise genome modification of agrobacterium may refer to Figure 1B. The specific steps are as follows.

*Step 1. Vector Construction.* Based on the pSG-SacB05 backbone vector, construct the agrobacterium modification tool plasmid with the lengths of upstream and downstream homologous arms larger than 400bp;
*Step 2. Transformation.* Transform the tool plasmid into the competent cells of agrobacterium by the freeze-thaw method, and coat the transformed product onto LB solid plates containing 25 mg/L rifampicin, 40 mg/L streptomycin, and 40 mg/L kanamycin. The plates were inverted and cultured at 28 °C for 3–5 days;
*Step 3. Induced modification.* Select several kanamycin resistant clones and inoculate them into LB liquid medium (pH = 5.5) containing 20 mg/L kanamycin and 300 μM acetosyringone. Incubate at 25 °C, 200 rpm for 3 days;
*Step 4. Optional step.* Inoculate the above bacterial solution at a ratio of 1:50 into fresh LB liquid medium (pH = 5.5) containing 20 mg/L kanamycin and 300 μM acetosyringone, and culture at 25 °C, 200 rpm for 3 days; (Extend the modification time to increase the modification efficiency)
*Step 5. Positive test for bacteria culture.* Use P1F, P1R, P2F, and P2R primers for dual PCR detection. If the modifications occurred at both the upstream and the downstream with specific electrophoretic bands, proceed with subsequent operations; Otherwise, repeat optional step 4 or restart a second culture after self-examination;
*Step 6. Bacteria isolation.* Take 1–2 μL bacterial solution above and dilute it 200 times with sterile water. Take 2μL, 10μL, and 50 μL of the diluted bacterial solution, respectively, and coat them onto LB solid plates containing 10% sucrose, 25 mg/L rifampicin, and 40 mg/L streptomycin. Inverted and cultured at 28 °C for 3–5 days;
*Step 7. Positive test for bacterial colony.* Select monoclonal colonies and use P1F, P1R, P2F, and P2R primers for dual PCR detection. Agrobacterium successfully modified should result in two specific bands;
*Step 8. Tool plasmid elimination.* Take 1–2 μL positive bacterial solution sample above and dilute it 100 times with sterile water. Take 1 μL, 10 μL, and 100 μL of the diluted bacterial solution, respectively, and coat them onto LB solid plates containing 10% sucrose, 25 mg/L rifampicin, and 40 mg/L streptomycin. Inverted and cultured at 28 °C for 3–5 days;
*Step 9. Retest for confirmation.* Select monoclonal colonies and use P1F, P1R, P2F, and P2R primers for dual PCR tests. Agrobacterium successfully modified should result in two specific bands;
*Step 10. Tool plasmid residue detection.* Perform dual PCR detection on positive strains above using SacB-64F, SacB-484R, P1F, and P2R primers. Select SacB negative (with no 458bp band), recover and perform Sanger sequencing on the amplified product of P1F and P2R primers to confirm the modification result;
*Step 11. Sanger sequencing, Kanamycin sensitivity test and Strain preservation.* Select strains verified by Sanger sequencing with correct modifications at both the upstream and the downstream, and inoculate them into LB liquid medium containing 25 mg/L rifampicin + 40 mg/L streptomycin and LB liquid medium containing 40 mg/L kanamycin + 25 mg/L rifampicin + 40 mg/L streptomycin, respectively. Culture at 28 °C and 200 rpm for 4 days; If the strain is sensitive to kanamycin (after cultivation for 4 days, the culture containing 40 mg/L kanamycin is still clear and transparent, and the kanamycin-free culture is turbid), then mix the culture with an equal volume of 40% sterile glycerol and let them well preserved at −80 °C.

Tips: In Step 1, if large fragment insertion or deletion modifications are required, it is recommended that the length of the homologous arms on both sides should be expanded. And optional Step 4 is recommended to improve modification efficiency. Acetosyringone stock is dissolved in dimethyl sulfoxide and shell be added to the LB medium just before incubation. It should be noted that, unless otherwise specified, the pH of LB medium is adjusted to 7.0.

### Sequences of primers used in modification tests

P1F: TTC​CCA​TCT​GCC​ACG​ACG​A; P1R: CAA​TAT​GCG​GAA​CAC​CAC​GC;
P2F: CGG​TTC​GGG​TTC​CGC​CCT​G; P2R: GTGCCATCGGGAAACGT;
SacB-64F: CTA​CCG​CAC​TGC​TGG​CAG; SacB-484R: TGT​GGC​TGA​ACC​TGA​CCA​TTC;

## Results

**FIGURE 2:**
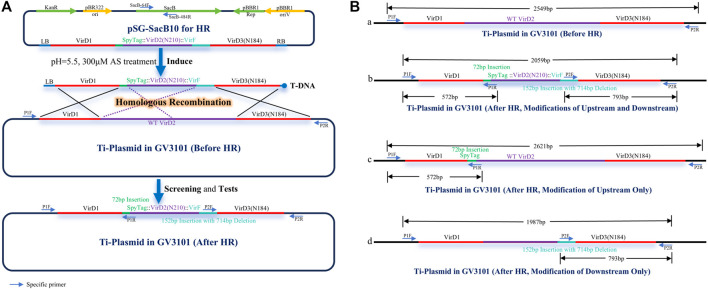
Schematic diagram for agrobacterium modification with pSG-SacB10 **(A)** and 4 possible modification results **(B)**. LB: left T-DNA repeat; RB: right T-DNA repeat.

The demonstration modification of agrobacterium strain GV3101 was conducted with the tool plasmid pSG-SacB10, which was designed to modify both the N-terminal and C-terminal of VirD2, which is located in the Ti-plasmid of GV3101. The 72bp insertion (SpyTag sequence) is flanked by the 487bp left homology arm (VirD1), and the 714bp deletion (C-terminal of VirD2) with 152bp insertion (VirF sequence) is flanked by the 574bp right homology arm (VirD3). The 630bp N-terminal sequence of VirD2 is reserved and might act as a middle homology arm, as shown in Figure 2A.

Theoretically, the modification of a specific gene at both the upstream and the downstream of it via homologous recombination will increase the complexity of the modification process. Taking the VirD2 as an example, after modification with the tool plasmid pSG-SacB10, 4 possible modification scenarios may arise: non-modification, modification at the upstream and the downstream, modification at upstream only, modification at downstream only, as shown in Figure 2B. These scenarios will correspond to different amplification products in PCR detection conducted with primers P1F, P1R, P2F and P2R.

The modification of GV3101 was conducted with the steps described in detail in Modification Steps. Five independent modification tubes were conducted in step 3 at pH = 5.5 with 300 μM acetosyringone, as this culture condition may be beneficial for the production of T-DNA. As a control test, 5 more independent modification processes were conducted at pH = 7.0 without acetosyringone.

### Modification at pH = 5.5 with 300 μM acetosyringone

**FIGURE 3:**
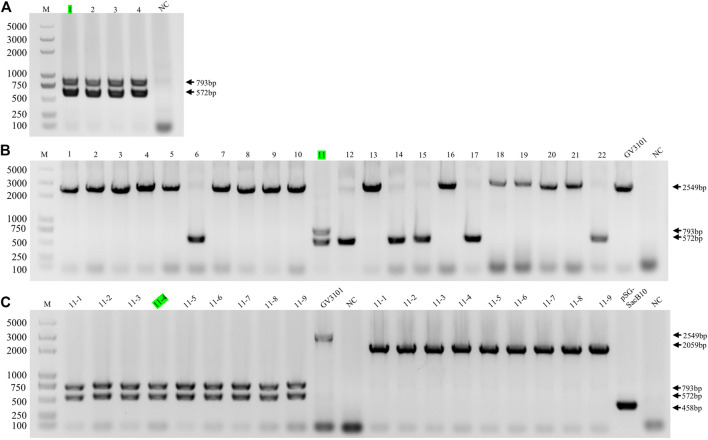
PCR detection results in step 5 **(A)**, step 7 **(B)**, step 9 [**(C)**, left] and step 10 [**(C)**, right] for modification products cultured at pH = 5.5 with 300 μM acetosyringone. NC indicates the negative control of PCR detection. The sizes of specific bands are indicated with black arrows. Lane numbers labeled with green background color indicates the sample used for subsequent experiments in the given spectrum.

After modification culture for 4 days, the PCR detection in step 5 showed that 4 modification cultures at pH = 5.5 with 300 μM acetosyringone had clear 572 bp and 793 bp specific electrophoretic bands, which indicated that the modification had occurred at the upstream and the downstream (one tube failed cultivation without detection), as shown in Figure 3A. Since there might be mixed modification products in the liquid culture condition. Monoclonal colonies originating from different modification tubes were tested again to further determine the type of modification. At the same time, 10% sucrose on a solid LB plate could kill those bacteria still containing pSG-SacB10, thus removing the tool plasmids.

It turned out that 13.6% (2/22, 2/22, 3/22, 6/22, 2/22 tested in step 7, respectively) monoclonal colonies had the 793bp bands, and 22.7% (4/22, 6/22, 4/22, 7/22, 4/22 tested in step 7, respectively) monoclonal colonies had the 572bp bands, 4.5% (1/22, 1/22, 1/22, 2/22, 0/22 tested in step 7, respectively) monoclonal colonies had the 793bp and 572bp dual-bands, refer to Figure 3B. Considering that the clone overlap or multicopy Ti plasmid may also lead to the generation of double bands. Three dual-band colonies were further separated into secondary monoclonal colonies. However, after a second separation in step 8, not all PCR-positive monoclonal colonies verified in step 7 bring out a positive dual-bands (1/9, 9/9, 9/9, in step 9, respectively). The residue of the SacB gene was also detected with specific primer pairs SacB64F and SacB484R. 27 monoclonal colonies, which survived on LB solid plates containing 10% sucrose, are all SacB negative (0/9, 0/9, 0/9, tested in step 10, respectively). Summary of these tests results and sample origin are listed in Supplementary Table S1.

Most of the secondary monoclonal colonies have specific dual-bands and the 2059bp band, which is consistent with the expected modification, as shown in Figures 2B, 3C. However, when a 793bp or a 572bp band occurred, the bands around 2000bp, which should have, usually do not exist or are very weak. Only one 1987bp event was detected in all of our tests, see Supplementary Figure S2C. This may be related to the lower amplification efficiency of long fragments in the same PCR reaction system.

**FIGURE 4:**
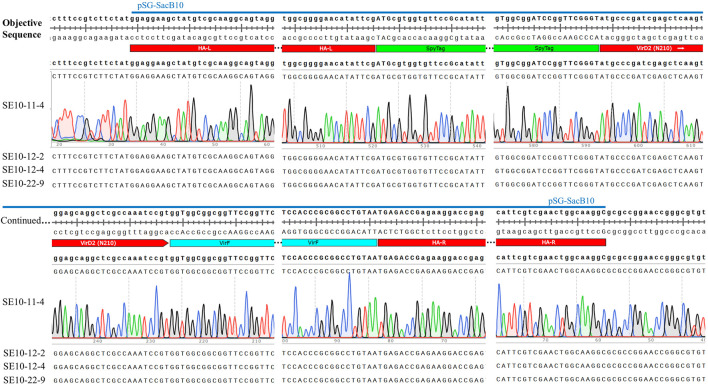
Sanger sequencing results of PCR products amplified from modified agrobacterium monoclonal colonies with P1F and P2R primer pair in step 11. Blue lines indicate the template sequences in the tool plasmid pSG-SacB10; on the contrary, unmarked sequences do not exist in pSG-SacB10.

To ultimately confirm the accuracy of the sequence of the modified product, four positive secondary monoclonal colonies, SE10-11-4, SE10-12-2, SE10-12-4, and SE10-22-9, were taken as candidate modified strains. These four modified bacterial strains were amplified again with primer pairs P1F and P2R, and all of them have the 2059bp bands, as shown in Supplementary Figure S2A. These amplification products were verified by Sanger sequencing, and all of them are 100% consistent with the objective sequence as designed. As displayed in Figure 4, sequences around the junctions of homology arm ends are displayed selectively. Besides, kanamycin sensitivity tests were performed to confirm that all these successfully modified colonies had no residual kanamycin resistance caused by the pSG-SacB10 plasmid. It turned out that they could not grow in LB medium containing 40 mg/L kanamycin; see Supplementary Figure S2B.

### Modification at pH = 7.0 without acetosyringone

**FIGURE 5:**
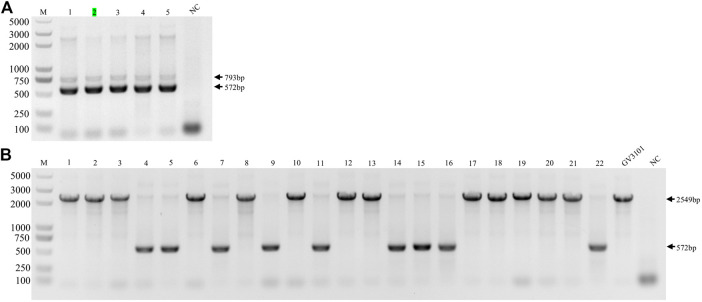
PCR detection results in step 5 **(A)**, step 7 **(B)** for modification products cultured at pH = 7.0 without acetosyringone. NC indicates the negative control of PCR detection. The sizes of specific bands are indicated with black arrows. Lane numbers labeled with green background color indicates the sample used for subsequent experiments in the given spectrum.

After modification culture at pH = 7.0 without acetosyringone for 4 days, the PCR detection in step 5 shown that 5 modification cultures had clear 572bp with weak 793bp bands, as shown in Figure 5A. This indicated that the modification of the downstream, which requires the long segment modifications, was not as effective as the upstream modification, which only has a 72bp insertion. After screening culture on 10% sucrose solid medium, no monoclonal colony with the 793bp band was found in 110 samples. And 39.1% (8/22, 9/22, 7/22, 9/22, 10/22 tested in step 7, respectively) of the monoclonal colonies had the 572bp bands. No effective downstream modification events were detected; refer to Figure 5B.

## Discussion

It can be seen that there are significant differences in the effect of gene modification under different pH values and whether acetosyringone is existent or not. Under the culture conditions of pH = 5.5 and containing 300 μM acetosyringone, we successfully obtained monoclonal colonies with the expected modification sequence. These colonies not only passed PCR detection, but also Sanger sequencing verification, and the target sequence was 100% consistent with the designed sequence. In addition, kanamycin sensitivity testing also confirmed that there was no residual kanamycin resistance caused by the pSG-SacB10 plasmid in these successfully modified colonies. Prior to this, we have tried to import some other tool plasmid, such as pSG-SacB01 (refer to Supplementary Figure S1), which lack the pBBR oriV, pBBR Rep, left T-DNA repeat and right T-DNA repeat sequences by freeze-thaw method for many times. However, no kanamycin resistant agrobacterium tumefaciens was obtained. Thus, the traditional modifications conduct with absolutely absence of T-DNA was missing. In another word, the electroporation method or three parent mating method is necessary to obtain integrated plasmids outcome in the traditional modifications of agrobacterium.

In contrast, the effect of gene modification was quite different under the culture conditions of pH = 7.0 and without the addition of acetosyringone. Although the liquid culture showed a weak 793 bp band, indicating a certain degree of modification in the downstream region, on the screening medium, no monoclonal colonies with a 793 bp band were detected, which further indicates the difficulty of long fragment modification at the downstream. For culture conditions of pH = 5.5 with 300 μM acetosyringone versus pH = 7.0 without acetosyringone, the upstream modification of VirD2 shifted from 22.7% to 39.1%, with the downstream modification of VirD2 shifted from 13.6% to zero. This opposite trend indicates that the modification mechanism of the small fragment may be very different from that of the long fragment. Since the pSG-SacB10 is a medium copy number plasmid driven by pBBR1 oriV in agrobacterium, the sequence of pSG-SacB10 itself may be enough for effective short fragment recombination but not long fragment modification.

Acetosyringone and pH have a significant impact on the infection efficiency of *Agrobacterium tumefaciens*. Acetosyringone can enhance the transformation effect by effectively inducing Vir gene expression (Gelvin, 2003; Tzfira and Citovsky, 2006), promoting T-DNA processing (Tzfira and Citovsky, 2006), and enhancing host sensitivity for agrobacterium (Jung et al., 2015). Weakly acidic conditions, such as pH from 5.2 to 5.8, is a key environmental signal for the production and transfer of agrobacterium T-DNA, which affects the transformation efficiency by regulating Vir gene expression (Stachel and Zambryski, 1986), enhancing bacterial attachment (Winans, 1992), and is beneficial for the stability of the T-DNA complex (Citovsky et al., 1992). As for agrobacterium itself, acetosyringone with pH = 5.5 are favorable conditions for the formation and stability of its endogenous T-DNA. Given that the single-strand DNA is a better homologous recombination template than the double-strand DNA (Tang et al., 2023), the single-strand T-DNA in agrobacterium induced by acetosyringone with pH = 5.5, should be the core reason for the improved efficiency of homologous recombination of long fragments at the downstream of VirD2.

In our prototype method for agrobacterium modification, with higher concentrations of antibiotics such as 50 mg/L streptomycin with 50 mg/L kanamycin, we have also successfully modified the C-terminal of VirD2 in GV3101 using pSG-SacB06-2G and pSG-SacB09 at an efficiency of less than 1%, see Supplementary Figure S3. Including these two cases of modification, 9 monoclonal colonies have been confirmed with Sanger sequencing, all of them are 100% consistent with the expected sequence as designed. This means that the accuracy of this method is quite good, although the efficiency is not as high as expected.

High concentrations of streptomycin or kanamycin may be a reason for low modification efficiency, as streptomycin or kanamycin has adverse effects on the expression of Vir genes (Gelvin, 2003; McCullen and Binns, 2006). More importantly, the commonly used Agrobacterium strains in plant genetic transformation, including GV3101, contain the function-less recA mutation, which is a key enzyme in its DNA repair mechanism (Attai et al., 2017; Pennetti et al., 2025). The absence of recA is more likely to result in low modification efficiency for this simplified modification method. In our future work, the introduction of functional recA will be crucial for further efficiency enhancement and host range expansion of this method.

## Conclusion

In this study, we successfully constructed the plasmid pSG-SacB05, which serves as a powerful tool for precise genome modification in agrobacterium. Through a series of well-defined modification steps, including the construction of a modification tool plasmid with adequate homologous arms, transformation into competent agrobacterium cells, selection of resistant clones, PCR detection, and Sanger sequencing, we achieved efficient and specific modifications at both the upstream and the downstream regions of the target genome at VirD2. Although the efficiency of this modification method is relatively low, the accuracy is very high. Considering the rapid growth ability of bacteria, this method can meet the practical applications. Overall, our study provides a straightforward and reproducible protocol for genome modification in agrobacterium via induced endogenous T-DNA, which can be referenceable to various genetic engineering and biotechnological applications.

## Data Availability

The original contributions presented in the study are included in the article/Supplementary Material, further inquiries can be directed to the corresponding author.
