# Supplementary material for: Precise genome modification of agrobacterium based on one-step homologous recombination via endogenous transfer DNA
Source: Acta Biochim Pol. 2026 May 25;73:16599. doi: 10.3389/abp.2026.16599 (PMC13251367; doi:10.3389/abp.2026.16599)
Supplement: Supplementary file 1 [file DataSheet1.docx]

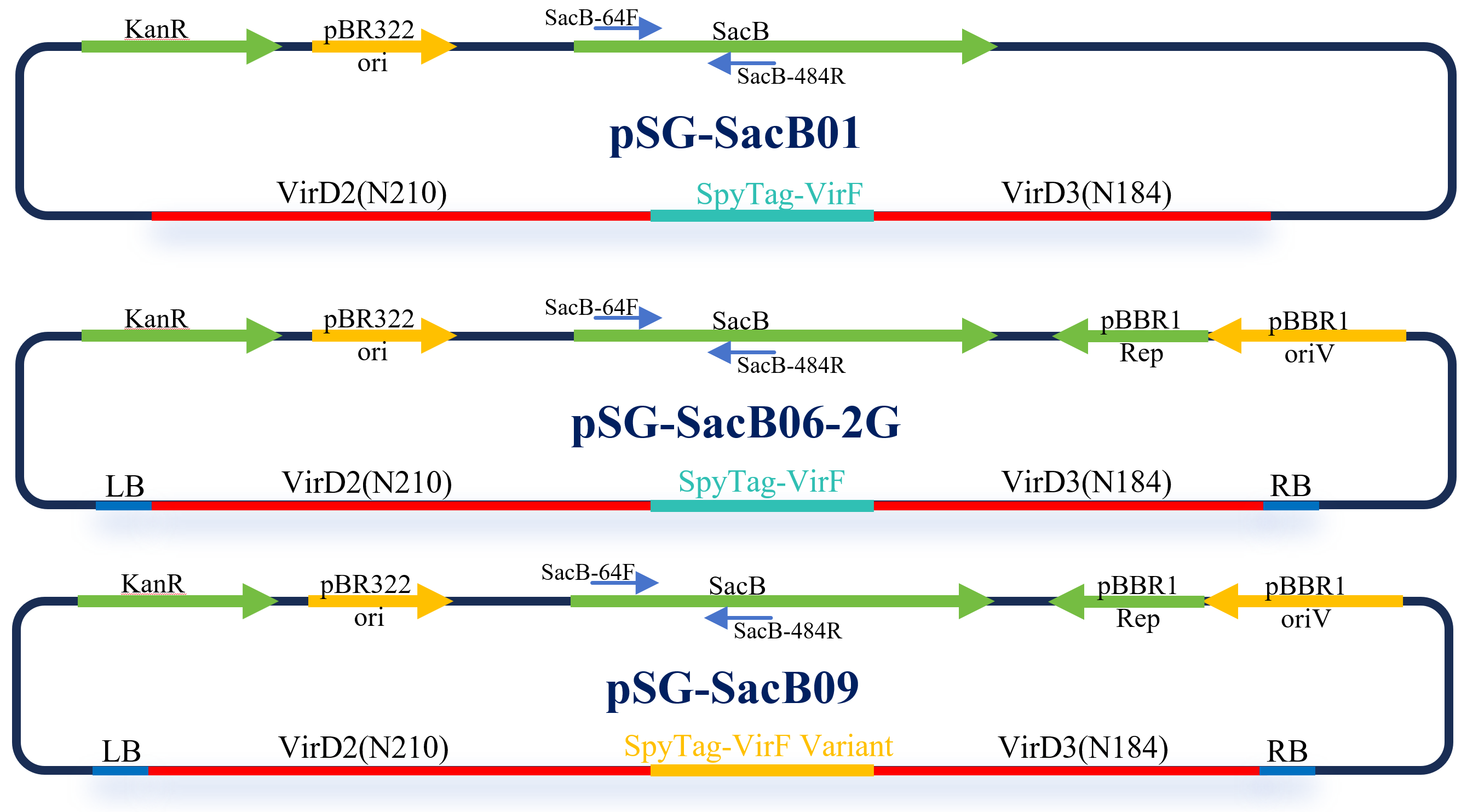
 **Figure S1.** Schematic diagram for plasmids pSG-SacB01, pSG-SacB06-2G and pSG-SacB09.


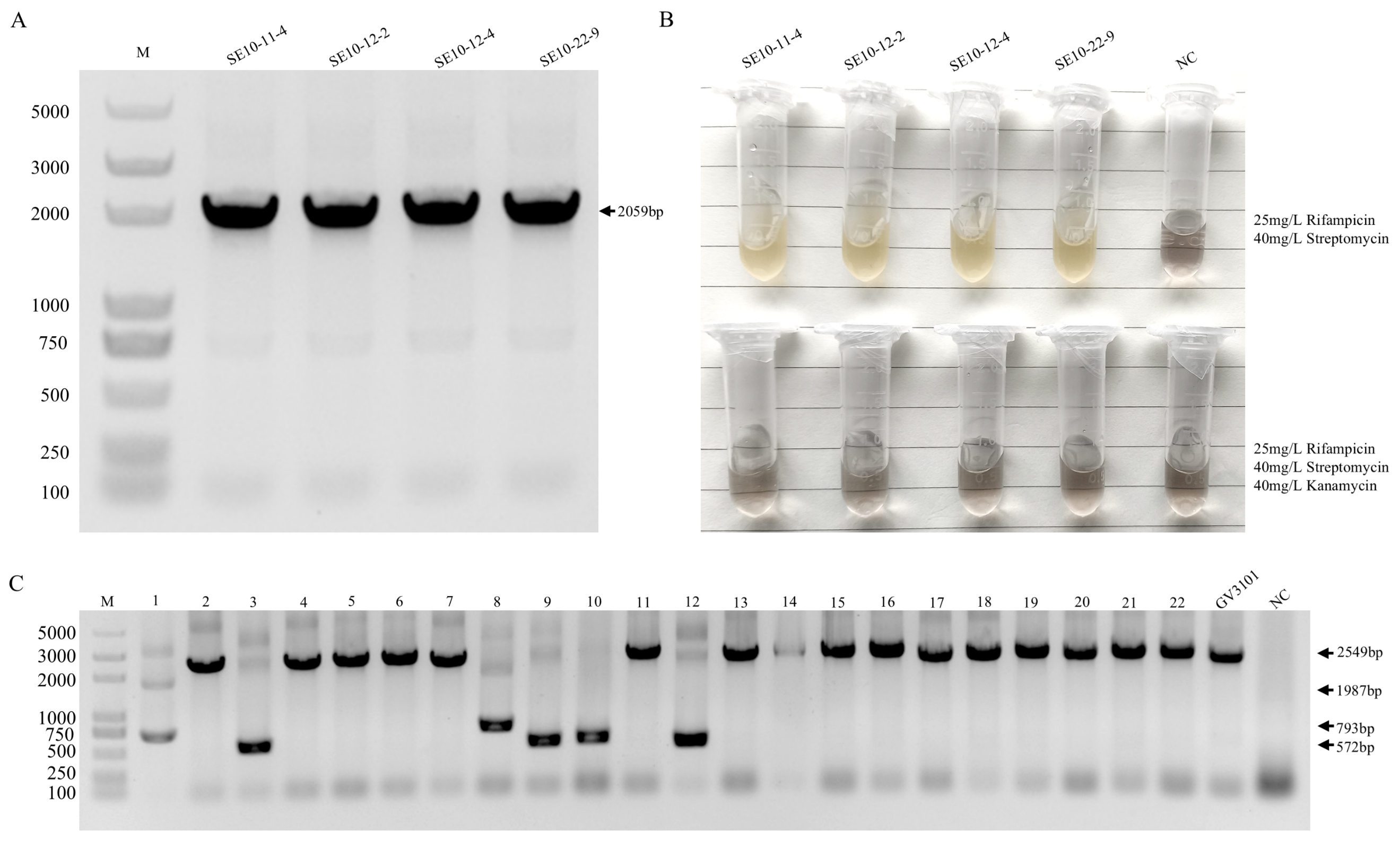


**Figure S2.** Supplementary results of several tests. A: Amplified DNA fragments used for Sanger sequencing; B: Results of kanamycin sensitivity test. C: Another PCR detection results in step 7 for monoclonal colonies originate from products cultured at pH=5.5 with 300μM acetosyringone.


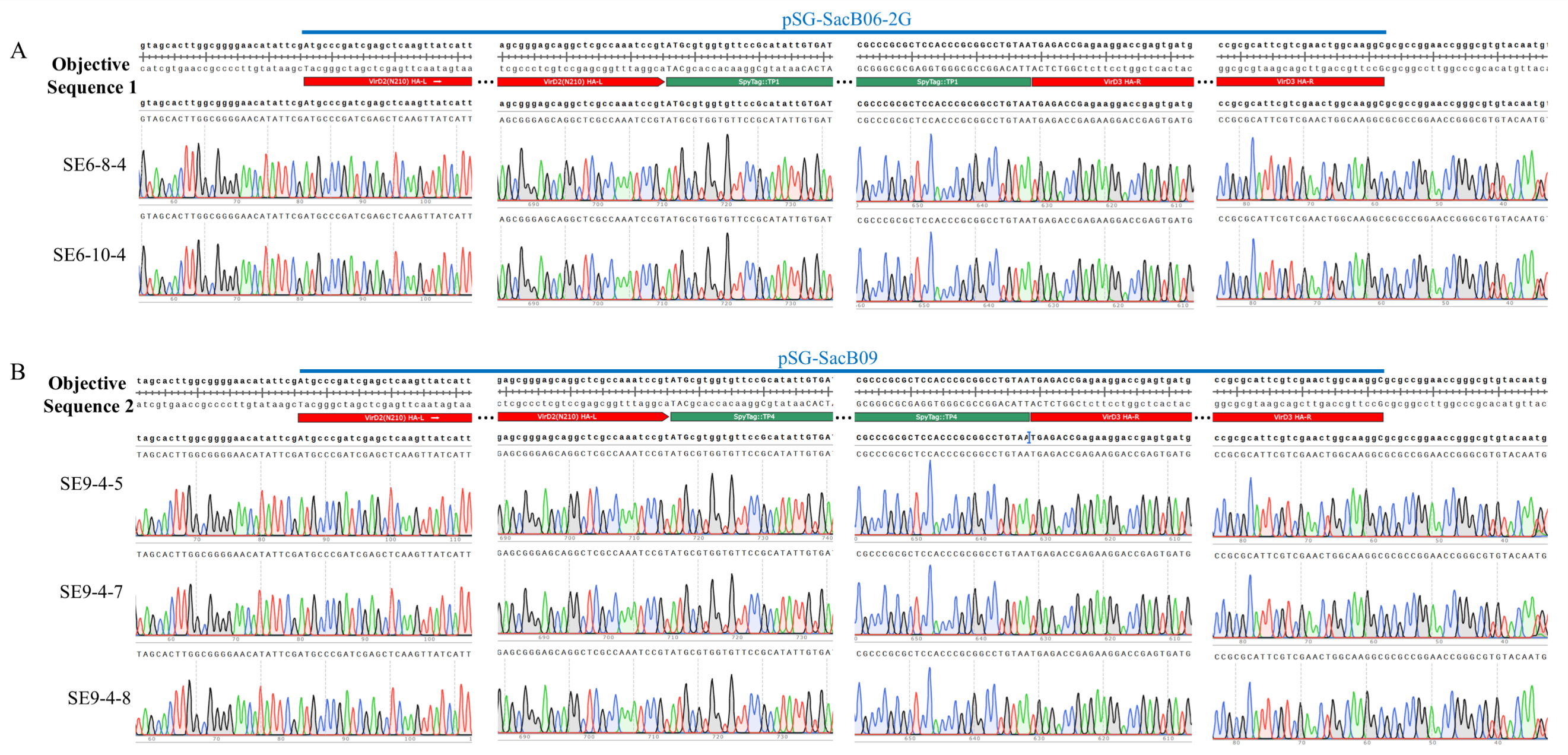


**Figure S3.** Sanger sequencing results of PCR products amplified from modified agrobacterium monoclonal colonies via the prototype method. Blue lines indicate the template sequences in the tool plasmid pSG-SacB06-2G (A) or pSG-SacB09 (B); on the contrary, unmarked sequences do not exist in the corresponding tool plasmid.

**Table S1.** Summary of tests results and sample origin for modifications with pSG-SacB10

| **Results** | **Steps** | | | | | | | |
| --- | --- | --- | --- | --- | --- | --- | --- | --- |
| **Modification Condition** | **Step 5** | | **Step 7** | | **Step 9** | | **Step 10** | **Step 11** |
|  | **Tube No.** | **Negative/ Positive** | **Positive Rate** | **Positive No.** | **Positive Rate** | **Positive No.** | **Positive Rate** | **Sanger Sequencing sample** |
| pH=5.5 with 300μM acetosyringone | Tube 1 | P | 1/22 | No. 12 | 9/9 | No. 1-9 | 0/9 | 12-2,12-4 |
|  |  |  | 1/22 | No. 11 | 9/9 | No. 1-9 | 0/9 | 11-4 |
|  | Tube 2 | P | 1/22 | No. 22 | 1/9 | No. 9 | 0/9 | 22-9 |
|  | Tube 3 | P | 2/22 | No. 3&16 | ND | NA | NA | NA |
|  | Tube 4 | P | 0/22 | NA | NA | NA | NA | NA |
|  | *Tube 5 | ND | NA | NA | NA | NA | NA | NA |
| pH=7.0 without acetosyringone | Tube 1 | WP | 0/22 | NA | NA | NA | NA | NA |
|  | Tube 2 | WP | 0/22 | NA | NA | NA | NA | NA |
|  | Tube 3 | WP | 0/22 | NA | NA | NA | NA | NA |
|  | Tube 4 | WP | 0/22 | NA | NA | NA | NA | NA |
|  | Tube 5 | WP | 0/22 | NA | NA | NA | NA | NA |

N: Negative; P: Positive; WP: Weak Positive; ND: Not Detected; NA: Not Applicable. * Indicate sample contamination.
